# Construction of a Zebrafish Model of Cardiac Hypertrophy Caused by ATIC Gene Deletion and Preliminary Exploration of Aerobic Exercise Improvement

**DOI:** 10.3390/ijms262110249

**Published:** 2025-10-22

**Authors:** Tianle Yang, Zhilong Zhang, Shuaiwang Huang, Mengchao Cui, Siyuan Liu, Meng Ding, Wenzhi Gu, Boyu Yang, Lan Zheng

**Affiliations:** 1 Hunan Key Laboratory of Physical Fitness and Sports Rehabilitation, Hunan Normal University, Changsha 410012, China; tianleyang@hunnu.edu.cn (T.Y.); 202320152955@hunnu.edu.cn (Z.Z.); 202320152962@hunnu.edu.cn (S.H.); 201820151262@hunnu.edu.cn (M.D.); 201630172015@hunnu.edu.cn (W.G.); boyuyang@hunnu.edu.cn (B.Y.); 2 Laboratory of Sports Biomechanics, School of Physical Education, Liaocheng University, Liaocheng 252059, China; 2320050401@stu.lcu.edu.cn; 3 Heart Development Center, College of Life Science, Hunan Normal University, Changsha 410081, China; 202220142830@hunnu.edu.cn

**Keywords:** hypertrophic cardiomyopathy, CRISPR/Cas9, ATIC, zebrafish, aerobic exercise intervention, transcriptome analysis

## Abstract

Hypertrophic cardiomyopathy (HCM) is a relatively common global cardiac disease, usually inherited, with complex phenotypes, genetic features, and a natural history. In this study, we constructed atic^−/−^ zebrafish using the CRISPR/Cas9 gene-editing system and found that atic^−/−^ zebrafish hearts exhibited HCM symptoms, and atic^−/−^ zebrafish hearts showed progressive enlargement, eccentric hypertrophy, cardiomyocyte enlargement, and collagen fiber deposition. Echocardiography results also showed that compared with atic^−/−^ zebrafish hearts, in wild-type zebrafish hearts, the ejection fraction was significantly reduced, shortening fraction was reduced, and ventricular wall thickness was significantly increased. Meanwhile, aerobic exercise intervention in atic^−/−^ zebrafish showed that aerobic exercise effectively improved the symptoms of HCM and improved cardiac function in atic^−/−^ zebrafish hearts. Transcriptome sequencing results showed that aerobic exercise improved the symptoms of HCM in atic^−/−^ zebrafish hearts involving the calcium signaling pathway, Apelin signaling pathway and ECM–receptor interaction. The q-PCR results of key differential genes involved in these pathways further confirmed that aerobic exercise could bring beneficial effects to atic^−/−^ zebrafish. In conclusion, this study found that the loss of ATIC can lead to hypertrophic cardiomyopathy in zebrafish, and aerobic exercise intervention can effectively improve the hypertrophic pathological characteristics of atic^−/−^ zebrafish hearts, providing new intervention targets and effective lifestyle interventions for HCM.

## 1. Introduction

5-Aminoimidazole-4-carboxamide nucleotide formyltransferase/inosinate cyclase 5-aminoimidazole-4-carboxamide ribotide formyltransferase/adenosine monophosphate cyclohydrolase (ATIC) catalyzes the synthesis of purine from scratch. This is a key enzyme in the last two basic steps of the de novo purine synthesis (DNPS) pathway that converts 5-aminoimidazole-4-carboxamide ribonucleotides 5-Aminoimidazole-4-carboxamide ribotide (AICAR) to inosine monophosphate (IMP) [1,2]. Recent studies have shown that ATIC-associated DNPS are strongly associated with cancer cell proliferation [3,4,5], that defective ATIC function leads to neurodevelopmental disorders and aortic stenosis [6], and that ATIC promotes pulmonary artery smooth muscle cell proliferation in pulmonary vascular remodeling through the Ras signaling pathway [7]. Increased expression levels of purine synthesis genes, including ATIC, were observed in damaged blood vessels and atherosclerotic lesions in mice and humans, and modulation of ATIC levels reduced pathological symptoms in atherosclerotic and restenosis models in mice [8]. These studies suggest that ATIC may regulate many pathological mechanisms in disease processes [9,10,11,12,13,14,15], but whether ATIC is involved in the pathogenesis of hypertrophic cardiomyopathy has not been identified.

Hypertrophic cardiomyopathy (HCM) is a genetically heterogeneous myocardial disease with a diverse natural course characterized by asymmetric thickening of the left ventricle and impaired diastolic function. Histopathological features include cardiomyocyte enlargement, cardiomyocyte disarray, and myocardial fibrosis [16,17]. Although these characteristics may lead to significant cardiac symptoms, many young patients with HCM are asymptomatic or have mild symptoms. HCM is a leading cause of heart failure (HF) and sudden cardiac death [18], so uncovering the genetic background of HCM can enhance our understanding of molecular pathogenesis and advance gene-based diagnostic tests to identify high-risk individuals and provide potential targets for the development of HCM treatments [19]. In approximately 40% of patients with HCM, the causative gene has not been identified [20].

Recent studies have shown that scientific exercise, as a non-drug intervention strategy, has significant effects in improving pathological myocardial hypertrophy, and exercise training can be used as a tool to discover new targets for heart failure treatment [21,22,23,24]. Aerobic exercise provides a relatively safe and significant improvement in cardiovascular disease. Studies have shown that aerobic exercise training can effectively reverse cardiogenic left ventricular dysfunction and induce anti-cardiac remodeling [25]. Regular aerobic exercise training provides blood pressure protection, improves myocardial function, and may reverse pathological cardiac hypertrophy [26].

In this study, we employed CRISPR/Cas9 to generate an “atic”-null zebrafish line and comprehensively evaluated whether the mutant recapitulates key hypertrophic cardiomyopathy (HCM)-like phenotypes. We further determined the therapeutic efficacy of aerobic exercise training and elucidated the underlying molecular mechanisms. Although rodents are physiologically closer to humans, their long reproductive cycle, high husbandry costs, and low throughput preclude large-scale intervention screens. Zebrafish (*Danio rerio*), a transparent vertebrate with rapid cardiac development, exhibits strong evolutionary conservation in Ca^2+^ handling, contractile-protein composition, and metabolic pathways relative to the human heart, making it an ideal model for early mechanistic studies of inherited cardiomyopathies. Consequently, the exercise-induced benefits observed in *atic*-deficient zebrafish hold high translational potential and may offer novel non-pharmacological strategies for human HCM.

## 2. Results

### 2.1. Construction of Zebrafish Lines with Atic Knockout

**Figure 1:**
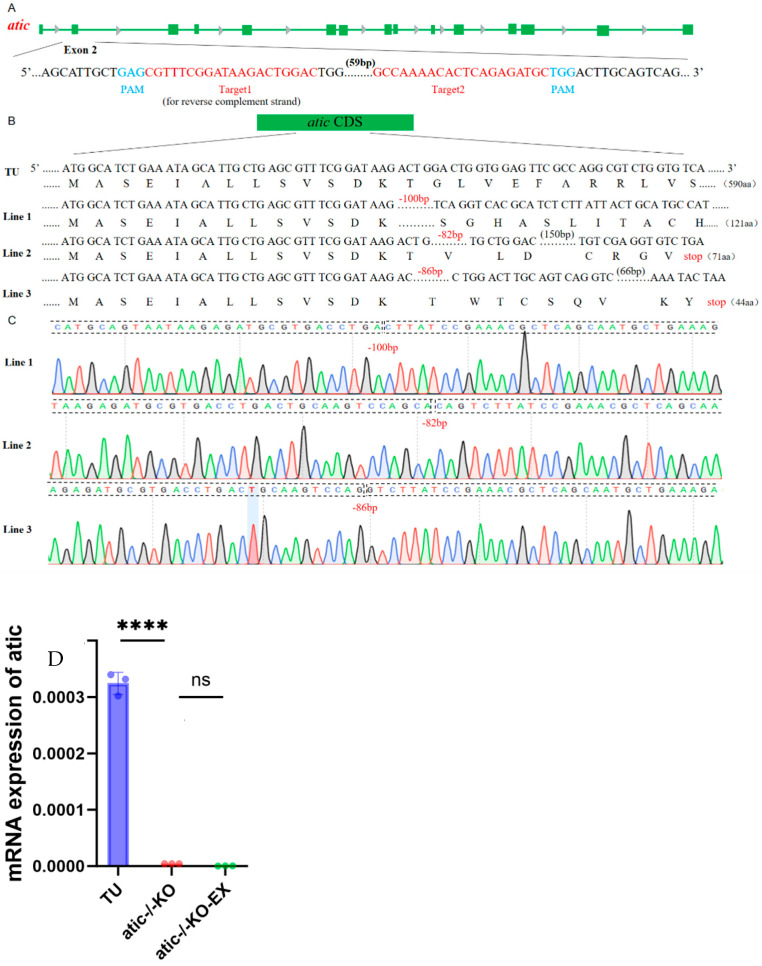
Schematic diagram of atic gene knockout in zebrafish. (**A**) Schematic diagram of sgRNA targeting for atic gene knockout. The green horizontal line represents the genomic DNA of atic, the green rectangle represents the exon of atic, the red represents the target sequence, and the blue represents PAM. (**B**) Schematic diagram of the three heritable mutant alleles of atic resulting from gene knockout and their encoded protein sequences. Sequencing peaks of three mutant alleles of (**C**) atic, where A, G, C, and T are represented by green, black, blue, and red curves, respectively. (**D**) qRT-PCR to verify the expression level of atic^−/−^RNA in mutant lines, data are shown as mean ± standard error, **** *p* < 0.0001, ns: *p* > 0.05 (*n* = 3 TU, *n* = 3 KO, *n* = 3 EX).

To investigate the role of the atic gene in the hearts of adult zebrafish, we constructed an atic-knockout zebrafish line. A pair of small guide RNA (sgRNA) targeting sites located in the second exon of atic, target 1 and target 2, were designed. They were designed to utilize the CRISPR/Cas9 gene-editing system to introduce frameshift mutations into the coding sequence (CDS) of atic via nonhomologous end joining (NHEJ), resulting in translational errors and premature termination codons for atic. A transcription template for sgRNA targeting atic exon 2 was synthesized by PCR (primers used are shown in Figure S1). Transcription was performed in vitro using T7 polymerase and the product was purified. Cas9 protein and sgRNA were co-injected into freshly fertilized eggs of wild-type zebrafish. Chimeras from surviving embryos were mated with wild zebrafish and mutant alleles with partial deletion of the atic gene in offspring were sequenced. Three zebrafish lines with the atic frameshift mutant alleles were identified by Sanger sequencing and designated lines 1–3. Specifically, row 1 contains two separate deletions (100 bp); a single deletion of 82 bp is found in row 2; and, row 3 contains a deletion of 86 bp (Figure 1B,C). Heterozygote inbreeding of three mutant lines produced F2 homozygotes. Since the mutant homozygotes of the above three atic-knockout lines did not differ significantly from the wild type in appearance, the following experiments were completed in the first row. Translation was terminated due to frameshift mutations in the atic^−/−^gene to further confirm successful construction of the atic^−/−^line. The mRNA levels were detected. RNA was extracted from 5-month-old adult zebrafish hearts by RT-qPCR and then inverted into cDNA for detection. As is shown in Figure 1D, RNA was hardly expressed at 5 months of age after the atic gene knockout, indicating successful knockout. Finally, we compared the FPKM values of *atic* across the RNA-seq datasets; the results remain consistent with our previous findings (Supplementary Table S1).

### 2.2. Aerobic Exercise Improves Cardiac Hypertrophy Phenotype of Atic^−/−^ Zebrafish

**Figure 2:**
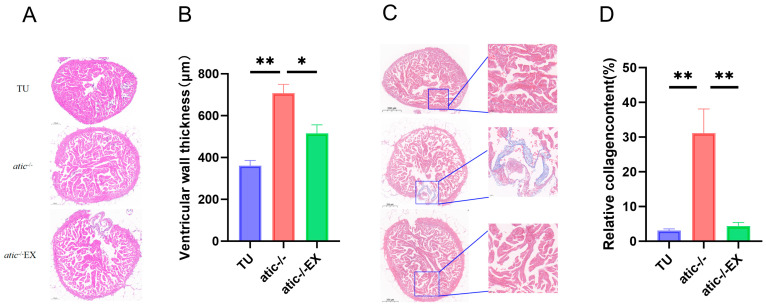
(**A**) H&E staining results of zebrafish adult heart; (**B**) H&E staining result analysis of zebrafish adult hearts (n = 3); (**C**) Masson staining results of zebrafish adult hearts; (**D**) Masson staining results analysis of zebrafish adult hearts (n = 3). * *p* < 0.05, ** *p* < 0.01. Scale bars: (**A**) 100 µm; (**C**) 200 µm.

The appearance of zebrafish hearts in this group is shown in Figure 2A. H&E staining showed progressive enlargement and eccentric hypertrophy of the atic^−/−^ zebrafish hearts compared to WT zebrafish, and swimming improved these symptoms (Figure 2B); Masson staining showed fibrosis deposition in the atic^−/−^ zebrafish hearts compared to WT zebrafish, while swimming reduced fibrosis deposition in the atic^−/−^ zebrafish hearts (Figure 2C). These results suggest that atic^−/−^ zebrafish hearts are consistent with hypertrophic heart pathology and that aerobic exercise improves these phenotypes. Please refer to Supplementary Figure S2 for the specific high-resolution image.

### 2.3. Aerobic Exercise Improves Cardiac Function in Atic^−/−^ Zebrafish

**Figure 3:**
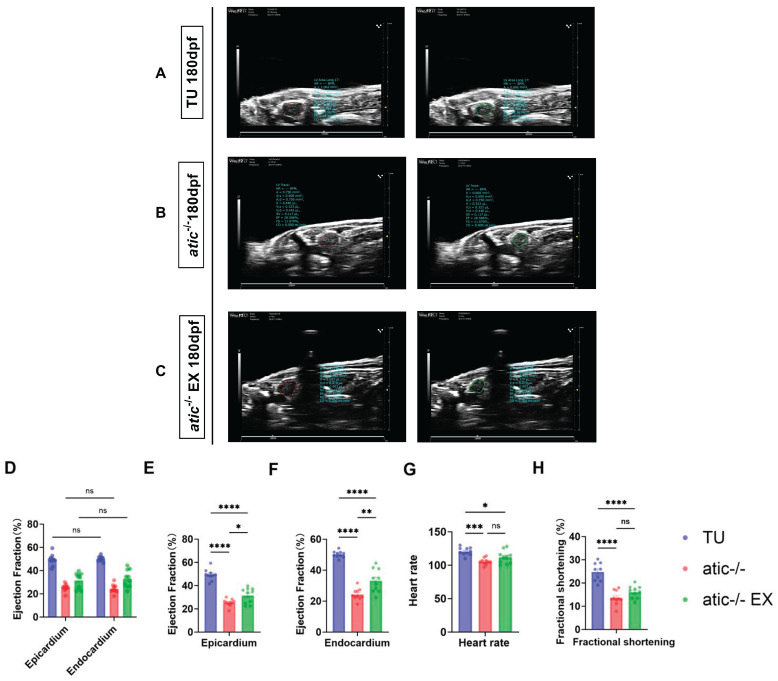
Cardiac function analysis of adult zebrafish (**A**–**C**). Note: Ventricular relaxation is depicted in red and ventricular contraction is depicted in green. This image is used to measure the cardiac function of zebrafish by tracing its ventricular epicardium. Each group consisted of 10 fish (n = 10) and each fish was analyzed in duplicate for three different cardiac cycles. (**A**–**E**) TU wild-type, atic^−/−^, atic + EX^−/−^ adult 3-month-old zebrafish echocardiography result analysis diagram; data are shown as mean ± standard error; note: (**D**): zebrafish echocardiography epicardial and endocardial ejection fraction result analysis comparison diagram (ns: *p* > 0.05); (**E**): zebrafish echocardiogram epicardial result analysis (* *p* < 0.05, **** *p* < 0.0001); (**F**): zebrafish echocardiography endocardial ejection fraction result analysis chart (** *p* < 0.01, **** *p* < 0.0001); (**G**): analysis graph of heart rate results of zebrafish TU wild-type, atic^−/−^, atic + EX^−/−^ groups (* *p* < 0.05, *** *p* < 0.001, ns: *p* > 0.05); (**H**): Analysis graph of shortening scores of zebrafish TU wild-type, atic^−/−^, atic + EX^−/−^ groups (**** *p* < 0.0001, ns: *p* > 0.05).

To further examine the cardiac function of the atic gene knockout in zebrafish, it was observed by echocardiography that the contractility of the atic-gene-knockout mutant heart was weaker than that of the wild type (Figure 3A–C). The analysis of the results showed that the gene-knockout mutant zebrafish had a significantly reduced shortening score compared with the wild-type zebrafish, while the shortening score increased with the aerobic exercise intervention (Figure 3H). Adult atic-gene-knockout zebrafish showed an abnormal heart function, and both epicardial and endocardial analysis methods were analyzed for the cardiac function index. The results showed that there was no significant difference between the two analysis methods (Figure 3D). The ejection fraction of wild-type zebrafish hearts was significantly lower than that of atic^−/−^ zebrafish hearts, while aerobic exercise intervention could increase the ejection fraction (Figure 3E,F). These results suggest that the aerobic exercise intervention enhanced cardiac function in the zebrafish with the atic gene knockout, resulting in partial improvement in cardiac hypertrophy.

### 2.4. Atic^−/−^ Zebrafish Heart Transcriptome Overview

**Figure 4:**
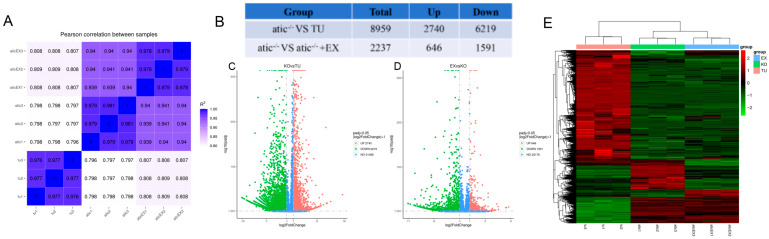
Correlation map of samples, volcano map, and heat map of differential gene expression. (**A**) Sample correlation plot; (**B**) differential gene summary statistical table; (**C**) atic^−/−^KO vs. TU group; (**D**) atic^−/−^EX vs. atic^−/−^KO group; (**E**) heat map; note: correlation of RNA-seq samples of 3-month-old wild-type zebrafish (TU), atic^−/−^, atic-EX^−/−^ groups; significantly up-regulated genes are indicated by red dots, significantly down-regulated genes are indicated by blue dots, and non-significantly different genes are indicated by gray dots. The abscissa and ordinate represent the negative correlation coefficient of expression level change. The larger the value, the more obvious the differential expression, and the higher the reliability of screening differential genes.

To further investigate the molecular mechanisms of the atic gene mutation leading to hypertrophic heart disease in zebrafish and exercise ameliorating atic^−/−^ hypertrophic heart disease in zebrafish, we performed RNA-seq on zebrafish hearts in each group. To ensure data accuracy and reliability, three tubes of 5-month-old zebrafish heart samples were tested in duplicate in all groups. According to the sample database sent, the correlation test chart of each group and each sample within the group was obtained (Figure 4A). The results show that the samples within the group have good repeatability and high correlation with each other; the samples between the groups have large differences, and the subsequent differential analysis is relatively reliable. After determining the reliability between samples, the expression differences in sequencing results were further analyzed statistically. The edgeR function package was used to successfully screen genes with significance level or *p*-value less than 0.05 and a multiple ratio of change greater than 1 (Figure 4B). Compared with the TU group, 8959 differential genes were enriched in the atic^−/−^ group, including 2740 up-regulated differential genes and 6219 down-regulated differential genes (Figure 4C). Compared with the atic^−/−^ + EX group, 2237 differential genes were enriched, of which 646 were up-regulated and 1591 were down-regulated (Figure 4D). These genes shown in the heat map of different groups (Figure 4E) have important guiding significance for subsequent bioinformatics analysis and research.

### 2.5. Biological Information Analysis of Aerobic Exercise Improving Cardiac Function of Atic^−/−^ Zebrafish

**Figure 5:**
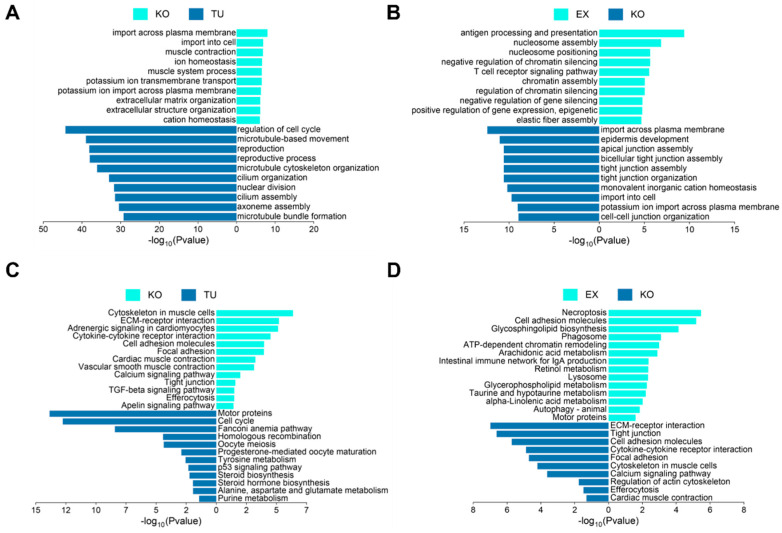
(**A**) atic^−/−^VS wild-type TU zebrafish GO (BP) analysis histogram; (**B**) atic^−/−^EX vs. atic^−/−^ group GO (BP) analysis histogram; note: green indicates KO enriched GO (BP) pathway in KO vs. TU group, blue indicates TU enriched GO (BP) pathway in KO vs. TU group; (**C**) atic^−/−^ vs. wild-type TU zebrafish KEGG analysis histogram; (**D**) atic^−/−^ EX vs. atic^−/−^ group KO KEGG analysis histogram; note: green indicates KO-enriched KEGG pathway in KO vs. TU group and blue indicates TU-enriched KEGG pathway in KO vs. TU group.

To further explore the underlying mechanisms of cardiac dysfunction caused by atic deficiency and the molecular mechanisms by which exercise improves cardiac function in atic^−/−^ zebrafish, we performed GO biological process enrichment analysis of differential genes. Muscle contraction, extracellular matrix organization, and extracellular structure organization were significantly enriched in the atic^−/−^ vs. TU group, and regulation of cell cycle, microtubule cytoskeleton organization, and cilium assembly were significantly enriched in the TU group (Figure 5A). The results of the atic^−/−^ + EX vs. atic^−/−^ group showed that antigen processing and presentation, chromatin assembly, and elastic fiber assembly were significantly enriched in the atic^−/−^ + EX group, and epidermis development, tight junction assembly, and cell–cell junction organization were significantly enriched in the atic^−/−^ group (Figure 5B). Similarly, we performed the Kyoto Encyclopedia of Genes and Genome (KEGG) enrichment analysis for the differential genes. The ECM–receptor interaction, calcium signaling pathway, and Apelin signaling pathway were significantly enriched in the atic^−/−^ vs. TU group; and, the cell cycle, P53 signaling pathway, and steroid biosynthesis were significantly enriched in the TU group (Figure 5C). Necroptosis, retinol metabolism, and autophagy were significantly enriched in the atic^−/−^ + EX group, and the ECM–receptor interaction, tight junction assembly, and calcium signaling pathway were significantly enriched in the atic^−/−^ group (Figure 5D).

### 2.6. To Verify the Expression of Genes Related to Cardiac Hypertrophy in Atic^−/−^ Zebrafish

**Figure 6:**
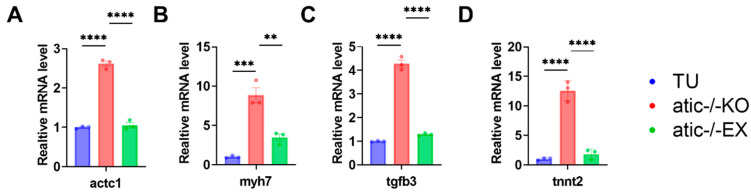
(**A**–**D**) qRT-PCR verification of the expression levels of actc1, myh7, tfgb3, tnnt2, and other genes in the heart after atic gene knockout. The data are displayed as mean ± standard error, ** *p* < 0.01, *** *p* < 0.001, **** *p* < 0.0001.

A fold change of ≥1 and *p*-value ≤ 0.05 were used as criteria to screen differentially expressed genes. Through screening of differentially expressed genes in transcriptome data, qPCR was used to verify the differential expression of actc1, myh7, tfgb3, and tnnt2. The results showed that the expression of cardiac hypertrophy factor was up-regulated in the atic-gene-knockout group compared with the wild-type group, while the expression of actc1, myh7, tfgb3, and tnnt2 was significantly down-regulated after 8 weeks of aerobic exercise intervention (Figure 6A–D).

### 2.7. To Verify the Molecular Mechanism of Exercise Improving Cardiac Function in Atic^−/−^ Zebrafish

**Figure 7:**
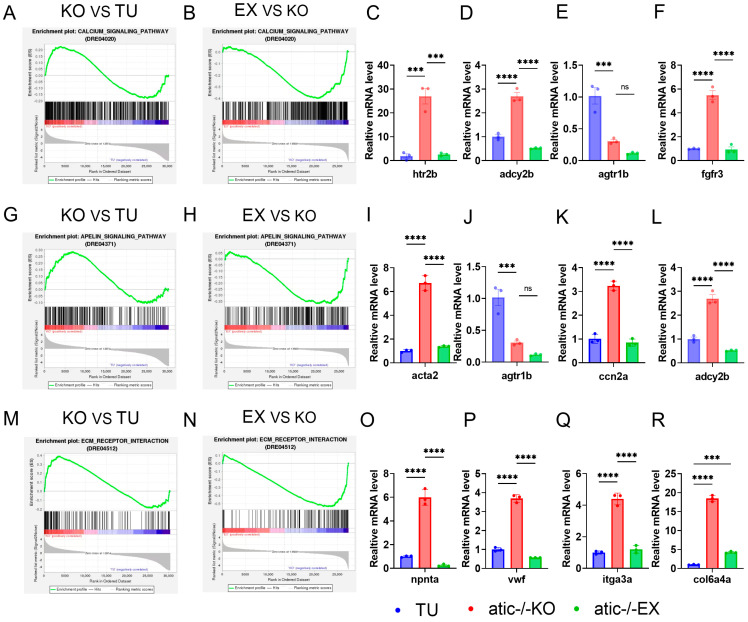
GSEA analysis results chart; (**A**,**B**) calcium signaling pathway GSEA analysis diagram, (**C**–**F**) qRT-PCR validation pathway of htr2b, agtr1b, fgfr3 gene expression level, *** *p* < 0.001, **** *p* < 0.0001, ns: *p* > 0.05; (**G**,**H**) Apelin signaling pathway GSEA analysis diagram, (**I**–**L**) qRT-PCR validation pathway of actca, agtr1b, ccn2a, adcy2b gene expression level, *** *p* < 0.001, **** *p* < 0.0001, ns: *p* > 0.05; (**M**,**N**): ECM–receptor interaction pathway GSEA analysis diagram, (**O**–**R**) qRT-PCR verification pathway of npnta, vwf, itga3a, col6a4a gene expression level, *** *p* < 0.001, **** *p* < 0.0001.

GSEA analysis found that the calcium signaling pathway (Figure 7A,B), Apelin signaling pathway (Figure 7G,H), and ECM–receptor interaction (Figure 7M,N) were significantly enriched and up-regulated in the atic^−/−^ vs. TU group, and significantly enriched and down-regulated in the atic^−/−^ + EX vs. atic^−/−^ group, indicating that exercise can improve the cardiac function of atic^−/−^ zebrafish. Further, using a fold change of ≥1 and *p*-value ≤ 0.05 as criteria to screen differentially expressed genes, we performed q-PCR verification of differentially expressed genes related to these three signaling pathways, which was consistent with GSEA.

## 3. Discussion

### 3.1. Effects of Exercise on Cardiac Function of Zebrafish with Atic Gene Knockout

This study demonstrates that aerobic exercise significantly improves the hypertrophic cardiomyopathy (HCM) phenotype in atic^−/−^ zebrafish, providing new insights into non-pharmacological interventions for inherited cardiomyopathy. Atic is a key enzyme in the purine de novo synthesis pathway, and its deficiency is closely related to metabolic disorders and cardiovascular diseases. We found that deletion of the atic^−/−^ gene leads to an imbalance in cardiac energy metabolism, leading to cardiac hypertrophy, fibrosis, and systolic dysfunction characteristics that are highly consistent with human HCM [27,28]. Notably, aerobic exercise intervention restored cardiac contractility, reduced ventricular wall thickness, and reduced fibrosis, suggesting that exercise-induced metabolic adaptation may offset the cardiac impairment associated with ATIC deficiency.

Mechanically, transcriptome analysis revealed aberrant regulation of key pathways in the atic^−/−^ heart, including Apelin signaling, extracellular matrix–receptor interaction (ECM–receptor interaction), and activation of calcium signaling. These pathways are closely related to myocardial remodeling, fibrosis, and calcium homeostasis. Exercise reversed these abnormalities, down-regulated genes associated with pathological hypertrophy (myh7, tnnt2), and restored pathways related to energy metabolism and cellular homeostasis. Among them, the AMPK/mTOR signaling axis—a central regulator of cardiac energy balance and autophagy—plays a key role in mediating exercise-induced cardiac protection [29]. The data are consistent with those of previous studies and suggest that AMPK activation enhances fatty acid oxidation and mitochondrial biosynthesis, thereby relieving metabolic stress on cardiomyocytes [30,31].

### 3.2. Transcriptome Characteristics of Motor-Regulated Atic^−/−^

RNA-seq analysis further elucidates the transcriptome remodeling effect of aerobic exercise on the atic^−/−^ zebrafish heart. After exercise intervention, necroptosis, retinol metabolism, and autophagy were significantly enriched in mutants, reflecting the enhancement of the cell repair mechanism. In contrast, pathways associated with ECM remodeling and calcium homeostasis dysregulation were inhibited, consistent with histologically reduced fibrosis and improved cardiac function. These findings are consistent with clinical observations that exercise training improves diastolic function and reduces fibrosis in patients with HCM.

Of particular importance was the down-regulation of Apelin signaling pathway expression after exercise. Apelin is a peptide hormone with cardioprotective effects, but it undergoes compensatory up-regulation in heart failure [32,33]. Inhibition of this pathway by exercise may indicate restoration of metabolic balance, thereby reducing pathological myocardial hypertrophy. Similarly, normalization of calcium signaling is associated with improved contractile efficiency, since calcium regulation is central to excitation–contraction coupling.

### 3.3. Enlightenment for HCM Treatment

This study highlights aerobic exercise as an effective strategy to mitigate the progression of HCM in ATIC-deficient models. By enhancing mitochondrial efficiency and activating adaptive signaling pathways, exercise compensates for deficiencies in purine synthesis, providing metabolic pathways for amelioration of cardiac dysfunction. This is consistent with emerging evidence that lifestyle interventions modulate inherited cardiomyopathy, challenging the conventional wisdom of relying solely on gene-targeted therapies.

However, this study has certain limitations. Short-term exercise protocols and zebrafish specific results need to be validated in mammalian models. Future long-term studies are needed to assess the continued benefits and age-dependent effects of exercise. In addition, the synergy of exercise with other treatments, such as AMPK activator drugs, remains to be explored.

In conclusion, aerobic exercise significantly ameliorated the HCM phenotype of atic^−/−^ zebrafish by correcting metabolic imbalances, inhibiting pathological signaling pathways, and enhancing mitochondrial toughness. These findings highlight the potential of exercise as a non-pharmacological intervention for HCM and related inherited cardiomyopathy. Future research should focus on translational validation and elucidation of the epigenetic mechanisms of exercise-induced cardiac protection.

## 4. Materials and Methods

### 4.1. Experimental Animals and Groups

The TU zebrafish strain was purchased from the National Zebrafish Resource Center (Wuhan, China) and cultured in our laboratory. It was maintained at a water temperature of 28 ± 1 °C and a light/dark cycle of 14/10 h. During the experiment, in addition to providing enough fresh shrimp for three groups of zebrafish at 9 a.m. and 6 p.m. each day, small amounts of shrimp were provided to all zebrafish before and after exercise. A total of 40 TU wild-type male zebrafish and 40 atic^−/−^mutant zebrafish of the same batch of 3 months old with similar body type were selected, and 40 zebrafish were then randomly selected for the control quiet group, with 20 atic^−/−^ zebrafish in the quiet group and 20 atic^−/−^ zebrafish in the exercise group. A total of 20 zebrafish were randomly selected for the swimming exercise intervention, and the quiet control group was not subjected to additional exercise intervention, and they were raised normally in the system. The atic^−/−^exercise group started their exercise at a water flow rate of 30%Ucrit, which was gradually increased to 50%Ucrit after 2 weeks, and they were then exercised at a water flow rate of 50%Ucrit for 6 weeks; each exercise period lasted 2 h, taking place 5 times a week, and the total exercise cycle was 8 weeks. At the end of the experiment, cardiac photography was performed using a UHF small animal imaging system; all aspects of this study were conducted according to the China Animal Welfare Guidelines and Experimental Protocol, approved by the Hunan Normal University Animal Experiment Management Committee (Changsha, China) (Approval No.: 2018/046) [34].

### 4.2. Aerobic Exercise Regimen

Swimming was as described above [35,36]. Before the start of the exercise experiment, the critical swimming speed of zebrafish was measured, and the critical swimming speed (Ucrit) was calculated by the Loligo^®^ system (#SW10600) (AutoResp™ v2.2.2, Loligo^®^ Systems, Tjele, Denmark; available online: https://loligosystems.com/downloads, accessed on 25 September 2025). In this experiment, a fish constant-temperature swimming training device (patent number: ZL2019 2 1672578.6) was selected, which was developed by the zebrafish experiment team of the Key Laboratory of Physical Fitness and Sports Rehabilitation of Hunan Province.

### 4.3. CRISPR/Cas9-Mediated Knockout of the Atic Gene

The zebrafish atic gene [37] was knocked out using the CRISPR/Cas9 gene-editing system. A PCR was performed using theP42250 plasmid as the sgRNA template to synthesize sgRNA transcripts. The reverse primer is sgRNA-R (universal R-terminal primer of target site; Figure S1), and the two forward primers containing T7 promoter and the target sequence are sgRNA-primer-F1 and sgRNA-primer-F2 (Figure S1), respectively. The sgRNA was synthesized by in vitro transcription using the Riboprobe^®^ System-T7 Transcription Kit (P1440, Promega, Madison, WI, USA). The sgRNA (final concentration: 20 ng/μL each) was co-injected into single-cell-stage fertilized eggs with Invitrogen TrueCut Cas9 v2 (A36499, Thermo Fisher Scientific, Waltham, MA, USA; final concentration: 300 ng/μL). The sgRNAs were designed to utilize CRISPR/Cas9 technology to introduce frameshift mutations into the coding sequence of atic through nonhomologous end joining, resulting in atic frameshift mutations that produce premature stop codons for protein translation. Surviving chimeric embryos were mated with WT zebrafish and progeny with the atic partial deletion mutant allele were sequenced. Atic knockout lines were genotyped using forward and reverse primers, atic F and atic R, respectively (Figure S1).

### 4.4. Hematoxylin–Eosin Staining

Zebrafish heart tissue was fixed in 10% formalin, embedded in paraffin, and sectioned. Before staining, sections were successively immersed in high to low concentrations of ethanol and washed with distilled water. The slides were then placed in hematoxylin solution for a few minutes and color separated in acid water and ammonia. After rinsing with running water, slides were stained with eosin solution, dehydrated, cleaned, and sealed.

### 4.5. Masson Trichrome Staining

Heart tissue was stained with hematoxylin iron solution (Sigma-Aldrich, St. Louis, MO, USA) for 5 min, washed 3 times in sterile deionized water, and stained with 0.7% Masson–Ponceau–acid fuchsin stain (Sigma-Aldrich) for 10 min. Heart tissue was washed with 2% glacial acetic acid, sectioned, and differentiated with phosphomolybdic acid solution for 4 min. Sections were then stained with 2% aniline blue stain (Sigma-Aldrich), dehydrated with graded concentrations of ethanol, washed with xylene, sealed with neutral gel, and observed and photographed under a light microscope (Olympus, Tokyo, Japan).

### 4.6. Echocardiographic Analysis

In this study, a Vevo3100 ultra-high resolution small animal ultrasound imaging system was used to detect the cardiac function of zebrafish after exercise; zebrafish were anesthetized in 0.2 mg/mL tricaine solution for 5 min, and then placed on the operating table made of sponge with their ventral side upward; the ultra-high resolution small animal ultrasound imaging system used a zebrafish ultrasound probe with a frequency of 48 MHz, mode of the B-Model, and a recording time of 300 ms [38,39].

### 4.7. Transcriptome Analysis

The 5-month-old TU group and atic^−/−^, atic^−/−^ zebrafish of the same period were randomly collected and those in the EX group were sent for sequencing. Three replicates of each group of samples were completed by Novogene (Beijing, China) Corporation, Ltd., using the IlluminaTruseq sequencer platform output in the fastq format. We downloaded the raw expression matrix from the Novogene Bioinformatics Technology Co., Ltd. customer portal, applied a threshold of *p* > 0.05 to exclude non-significant signals, and performed all subsequent data processing and downstream analyses (volcano plotting, GO, KEGG, and GSEA) through the company’s integrated online pipeline.

The volcanic map of the DEGs was drawn using three software packages: DESeq2 (version 1.57.1), DEGseq (version 1.56.0), and edgeR (version 4.0.1). Genes/transcripts with similar expression patterns may have similar functions or participate in the same metabolic process or cellular pathway. Based on the expression level of genes/transcripts in different samples, the distances between genes/transcripts or samples were calculated, and iterative methods were then used to cluster genes or samples. GO enrichment analysis was performed using Goatools software (https://github.com/tanghaibao/GOatools, version 0.6.9, accessed on 25 July 2023), and when corrected *p*-values (FDR) were <0.05, they were considered to be significantly enriched, and sufficient for GO function. KEGG pathway enrichment analysis was performed using R language. The principle of calculation was similar to that of the enrichment analysis of the GO function, with *p* < 0.05 as the cut-off criterion. Gene Set Enrichment Analysis was performed using clusterProfiler (version 4.2) and the MSigDB zebrafish gene sets (c2.cp.kegg.v7.5.1). Phenotype permutation = 1 000, minSize = 15, maxSize = 500. Significance: FDR < 0.25.

### 4.8. qPCR Detection

To detect gene expression, qPCR was used. Each group consisted of 3 biological replicates, each consisting of 5-month-old zebrafish heart tissues and 4 technical replicates. Total RNA extraction involved synthesis of cDNA using the Novozyme Universal RNA Extraction Kit (RC112-01, Vazyme, Dusseldorf, Germany) and PrimeScript RT Kit (RR 036A-1, Takara, Japan). Target gene expression was normalized using an internal control GAPDH. The qPCR was performed using a QuantStudio 5 real-time fluorescence quantitative PCR system (Thermo Fisher Scientific, Waltham, MA, USA) and QuantStudio design and analysis software (version 1.5.2). Table S2 details primers for target genes, including myh7, tnnt2, actc1, tgfb3, htr2b, adcy2b, agtr1b, fgfr3, acta2.ccn2a, npnta, vwf, itga3a, and col6a4a. Relative gene expression values were calculated using the ΔΔCT method.

### 4.9. Statistical Analysis

The statistical significance of qPCR and cardiac function test data was assessed using an unpaired *t*-test with statistical significance levels set to *p* < 0.05 for the results.

## 5. Conclusions

The present study elucidated the role of ATIC deficiency in the development of hypertrophic cardiomyopathy (HCM) in zebrafish and investigated the therapeutic potential of exercise intervention. The *atic*-gene-knockout zebrafish model exhibited characteristic HCM phenotypes, including cardiac enlargement, cardiomyocyte hypertrophy, and fibrosis, accompanied by impaired cardiac function. Exercise intervention significantly ameliorated these pathological features and improved cardiac function by modulating key signaling pathways such as the calcium signaling pathway, Apelin signaling pathway, and ECM–receptor interaction.

The findings highlight the importance of ATIC in maintaining cardiac homeostasis and suggest that exercise can serve as a non-pharmacological intervention for HCM. The molecular mechanisms underlying the beneficial effects of exercise involve the regulation of metabolic pathways and the inhibition of pathological signaling cascades. Future research should focus on validating these findings in mammalian models and exploring the long-term effects of exercise on HCM progression. Additionally, the potential synergistic effects of exercise with pharmacological interventions warrant further investigation. Overall, this study provides novel insights into the pathogenesis of HCM and underscores the therapeutic potential of exercise in managing this condition.

## Data Availability

The raw RNA-seq datasets generated during the current study are available in the NCBI Sequence Read Archive (SRA) under BioProject ID PRJNA1329260 (https://www.ncbi.nlm.nih.gov/bioproject/PRJNA1329260, accessed on 30 September 2025). All other data supporting the findings of this manuscript are available from the corresponding author upon reasonable request.

## Supplementary Materials

The following supporting information can be downloaded at: https://www.mdpi.com/article/10.3390/ijms262110249/s1.
