# Supplementary material for: Construction of a Zebrafish Model of Cardiac Hypertrophy Caused by ATIC Gene Deletion and Preliminary Exploration of Aerobic Exercise Improvement
Source: Int J Mol Sci. 2025 Oct 22;26(21):10249. doi: 10.3390/ijms262110249 (PMC12607423; doi:10.3390/ijms262110249)
Supplement: Supplementary file 1 [file ijms-26-10249-s001.zip › ijms-3833953-supplementary.pdf]

Figure S1:

A B

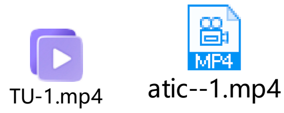

Analyze the cardiac function of adult zebrafish using an ultra-high frequency small animal imaging system.A): TU;(B): *atic*.

Table S1:

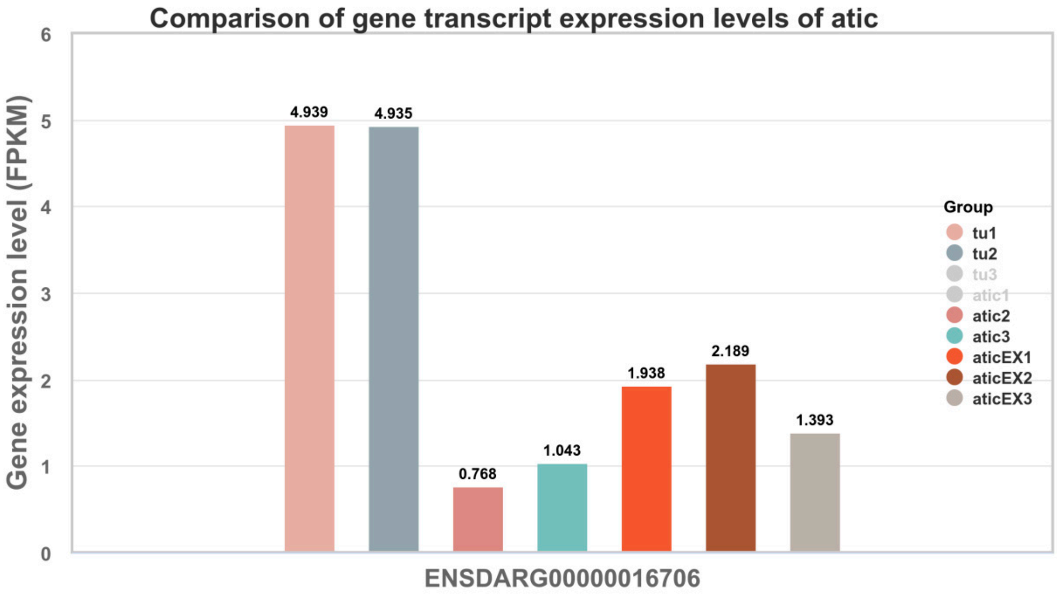

The author selected the fkpm values of the atic gene (Gene ID: ENSDARG00000016706) from the transcriptome data across all groups and conducted a comparative analysis.

Figure S2:

(A) : HE staining results of zebrafish adult heart;(B): Masoon staining results of zebrafish adult heart.

A

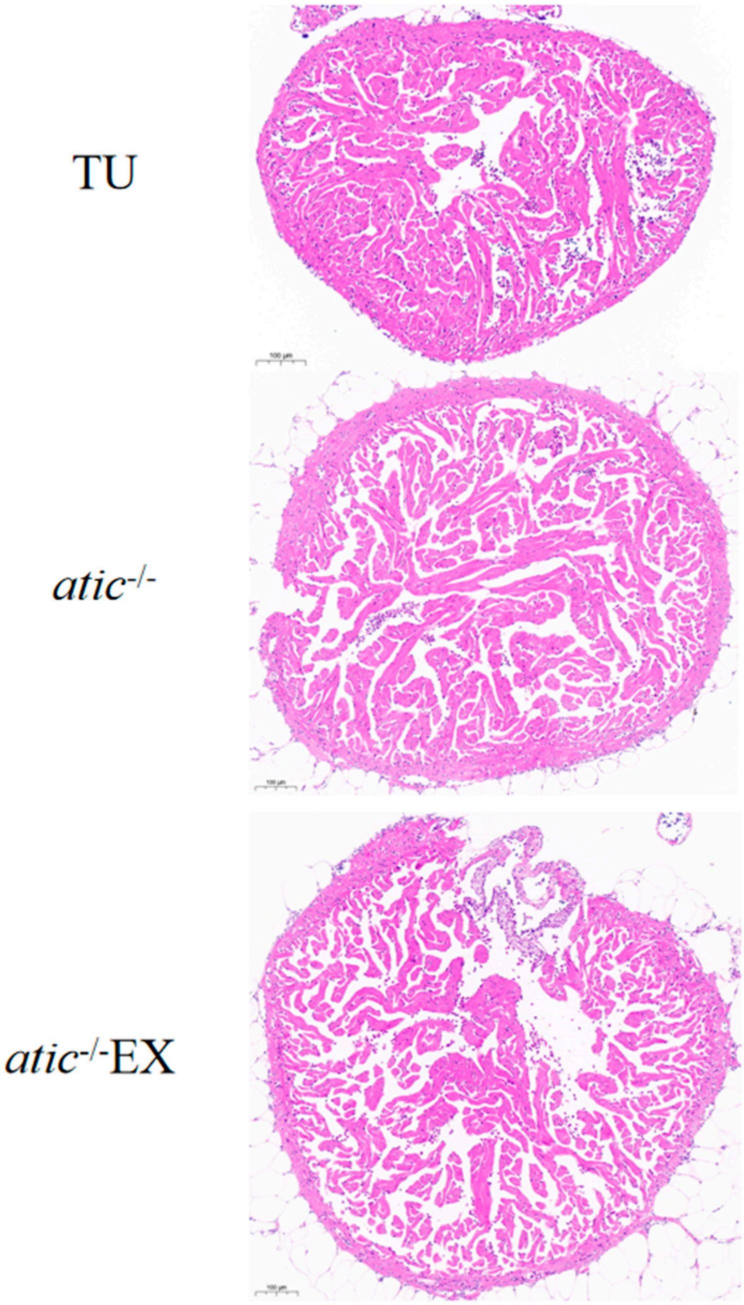

B

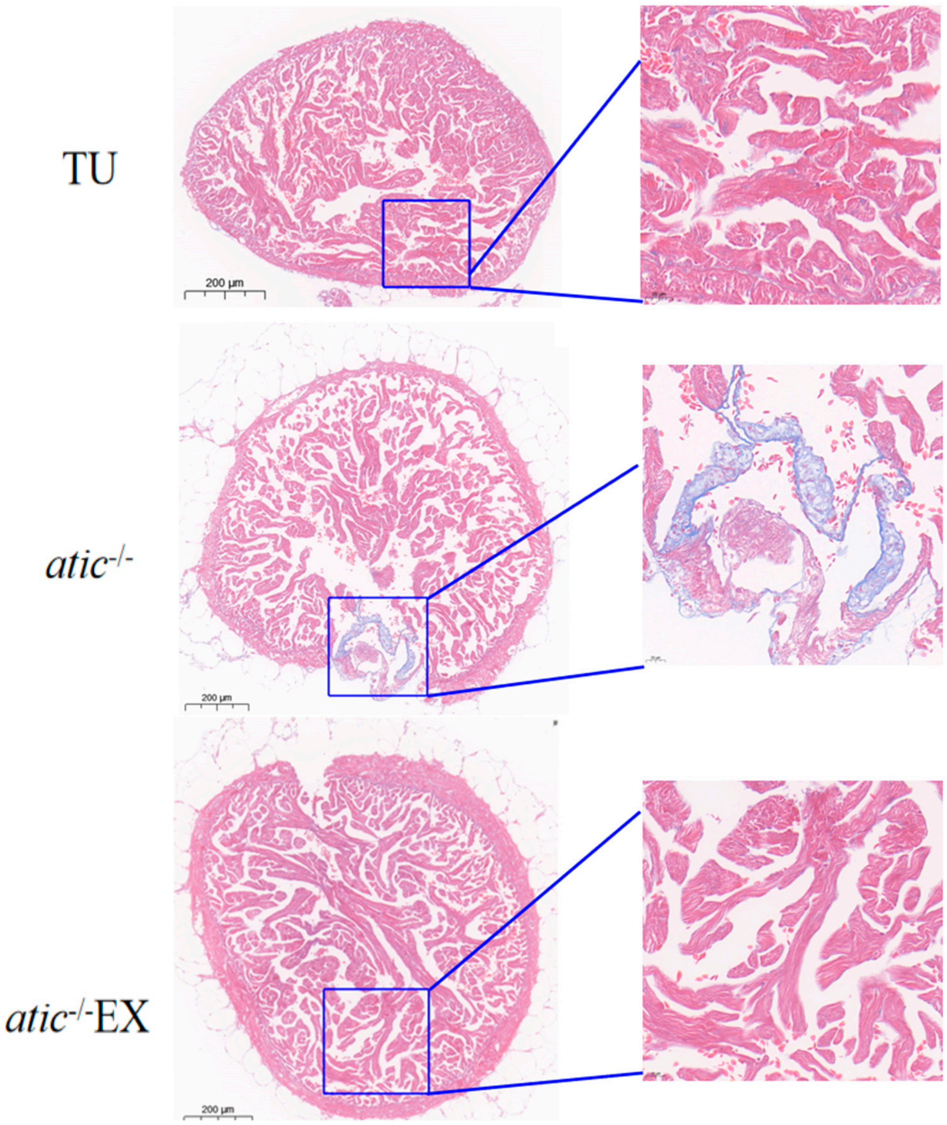

Table S2：

qPCR primers used for detecting atic knockout efficiency and cardiac-hypertrophy-related genes in zebrafish

Gene	Sequence (5'→3')
atic-F	TCGGGTTCTTGAGGAGGCTA
atic-R	AATTGTTGCCACGGTAACGC
myh7-F	CTTGGTGCACATCAGACAAGG
myh7-R	CTGGGGGTGAATGTCAGCTT
col6a4a-F	TGGCGAGTCCGAGGATGATGTC
col6a4a-R	GTTCTTCACTCCGAGGGCATACAC

tnnt2-F	GAAGGCCAGTGAAATGTGGC
tnnt2-R	CGACCTTTGGCACTCTGGT
agtr1b-F	ACCTTCCTTATCACGCTGCC
agtr1b-R	GCACCGGATGTACGATAGCA
fgfr3-F	AGGCAGGTAATGGAAATGCAGA
fgfr3-R	CAGCTTAATGCCGCCCATTC
adcy2b-F	GCAGTACAACTGTCAGCAAAGAA
adcy2b-R	GAGTCGTGGCTCTGTACTCTT
htr2b-F	CCTGGTCATTCTGGCAGTGT
htr2b-R	GGGGCCATGTGGAGTTGTAA
tgfb3-F	AGTCAGGTGCCCTACCAAGT
tgfb3-R	CAGTAGGGCAGGTCATTGTTTT
actc1-F	TGCGCTACAACTTTGAACCATC
actc1-R	ACGGGGTCTGCCAACAATAG
itga3a-F	ACCGCTTCTGTTCAGACCCT
itga3a-R	CGGAGTCCTTTGCGGTGAAA
acta2-F	GCGGTGTTCCCTTCCATAGT
acta2-R	AGGTCTCAAACATGATCTGGGT
ccn2a-F	AATGGTGTACCGCAGTGGAG
ccn2a-R	CTCCTCCTCTCTGTAAGCTGC
vwf-F	GTCAAAACATCACCCGCACC
vwf-R	AGTATGTGCTTCCCACAGGG
npnta-F	CAGCCCCTCTACGTCTTAACC
npnta-R	CCACACTCGTTCAGGTCTTG
